# H1N1 G4 swine influenza T cell epitope analysis in swine and human vaccines and circulating strains uncovers potential risk to swine and humans

**DOI:** 10.1111/irv.13058

**Published:** 2022-10-25

**Authors:** Swan Tan, Lenny Moise, Douglas S. Pearce, Constantinos S. Kyriakis, Andres H. Gutiérrez, Ted M. Ross, Justin Bahl, Anne S. De Groot

**Affiliations:** ^1^ Department of Infectious Diseases, College of Veterinary Medicine University of Georgia Athens Georgia USA; ^2^ Center for Vaccines and Immunology, College of Veterinary Medicine University of Georgia Athens Georgia USA; ^3^ EpiVax Inc. Providence Rhode Island USA; ^4^ Zoetis Inc., Veterinary Medicine Research and Development Kalamazoo Michigan USA; ^5^ Department of Pathobiology, College of Veterinary Medicine Auburn University Auburn Alabama USA; ^6^ Institute of Bioinformatics University of Georgia Athens Georgia USA; ^7^ Department of Epidemiology and Biostatistics University of Georgia Athens Georgia USA

**Keywords:** conservation, human, Immunoinformatics, influenza, swine, T cell epitope

## Abstract

**Background:**

Pandemic influenza viruses may emerge from animal reservoirs and spread among humans in the absence of cross‐reactive antibodies in the human population. Immune response to highly conserved T cell epitopes in vaccines may still reduce morbidity and limit the spread of the new virus even when cross‐protective antibody responses are lacking.

**Methods:**

We used an established epitope content prediction and comparison tool, Epitope Content Comparison (EpiCC), to assess the potential for emergent H1N1 G4 swine influenza A virus (G4) to impact swine and human populations. We identified and computed the total cross‐conserved T cell epitope content in HA sequences of human seasonal and experimental influenza vaccines, swine influenza vaccines from Europe and the United States (US) against G4.

**Results:**

The overall T cell epitope content of US commercial swine vaccines was poorly conserved with G4, with an average T cell epitope coverage of 35.7%. EpiCC scores for the comparison between current human influenza vaccines and circulating human influenza strains were also very low. In contrast, the T cell epitope coverage of a recent European swine influenza vaccine (HL03) was 65.8% against G4.

**Conclusions:**

Poor T cell epitope cross‐conservation between emergent G4 and swine and human influenza vaccines in the US may enable G4 to spread in swine and spillover to human populations in the absence of protective antibody response. One European influenza vaccine, HL03, may protect against emergent G4. This study illustrates the use of the EpiCC tool for prospective assessment of existing vaccine strains against emergent viruses in swine and human populations.

## 1 INTRODUCTION

The annual occurrence of influenza outbreaks causes considerable morbidity and mortality and poses a global public health challenge. The causative agent of these outbreaks is influenza A virus (IAV), although influenza B also contributes to some outbreaks. IAV infects a wide range of hosts including mammals and avian species. Pigs are one of the most important hosts given their susceptibility to a broader range of avian and human influenza viruses, and they are often the source of novel reassortant viruses from avian‐ and human‐origin strains. IAV infection of swine causes significant economic losses for commercial pork producers. In addition, swine IAV poses a threat to human health due to the potential for swine influenza A virus (swIAV) to spill over into the human population, as occurred in 2009 (for H1N1/pdm09).^1^, ^2^

After the outbreak of H1N1/pdm09 in human in 2009, the virus was reintroduced into pig herds around the world and reassorted with other swine influenza viruses, forming new swIAVs which spread within pig herds in the United States (US), Brazil, Europe (EU), Japan, China, and other countries.^3^, ^4^ Some of these reassortants harboring H1N1/pdm09 internal genes have gradually evolved and replaced previous strains of swine influenza, demonstrating antigenic drift due to genetic selection pressure exerted on H1N1/pdm09 and other strains worldwide.^5^ For example, multiple lineages of swIAV have been identified in pig herds in China, including classical swine H1N1, Eurasian avian‐like (EA) H1N1, H1N1/pdm09, triple‐reassortant (TR), and H3N2 lineages.

Influenza experts have been concerned about a new strain of swIAV, namely, the G4 genotype (G4), that is now dominating other strains of IAVs in Chinese swine populations, as reported by Sun et al.^6^ Emergent G4 is a reassortment product of EA H1N1 virus, bearing H1N1/pdm09 and TR‐derived internal genes. In the reassorted G4, the HA and NA genes are from the EA H1N1 lineage, and in particular, the HA gene falls within the 1C.2.3 lineage. The viral ribonucleoprotein (vRNP) genes and M gene are from the H1N1/pdm09 lineage, and the non‐structural (NS) gene is derived from the TR lineage.^6^ Given that as many as 20% of pork industry workers in China have been found to be seropositive for G4 antibodies, it appears that G4 has the potential to cross species barriers.^6^

Vaccine efficacy evaluation usually involves assessment of cross‐reactive influenza‐specific antibodies generated by exposure or vaccination. Seasonal vaccination in humans does not generate antibodies that protect against G4 (hemagglutination inhibition; see Figure 1C in Sun et al.^6^). In a separate study, monoclonal antibodies isolated from mice immunized with pandemic (A/California/07/09) hemagglutinin (HA) and a novel flu vaccine, computationally optimized broadly reactive antigen (COBRA) P1 HA, generated hemagglutination of G4 virus‐like particles in vitro.^7^ The relevance of this murine study to swine and human populations remains to be determined.

When cross‐reactive antibodies are not present, cross‐conserved T cell epitopes in IAV vaccines, and strains have been shown to play an important role in reducing morbidity and limiting the spread of IAV, even when vaccines and emergent strains are poorly matched.^8^, ^9^, ^10^ There is strong evidence that (1) T cell responses generated by previous influenza exposure cross‐reacts with novel IAV strains^11^ and (2) T cell responses are critically important for protection against IAV infection in both humans and swine.^10^, ^12^, ^13^ Thus, even in the absence of cross‐reactive antibody to G4, T cell cross‐reactivity might be protective.

Here, we apply an immunoinformatics tool to evaluate whether existing vaccines may have the capacity to prevent the spread of G4 in humans and swine. We developed a computational workflow that employs the Epitope Content Comparison (EpiCC) algorithm to measure the degree of epitope conservation between target vaccines and outbreak strains. In previous studies, we identified an EpiCC score that was correlated with protection in the absence of cross‐reactive antibody. We used the same approach to establish thresholds for protective efficacy for vaccines against circulating strains in this study. EpiCC's estimation of T cell epitope conservation between emerging viruses and vaccine strains may be useful as a potential surrogate measure of vaccine efficacy, in conjunction with other methods of pandemic risk assessment.

## 2 METHODS

### 2.1 Sequences and data processing

#### 2.1.1 Vaccine strains

The H1 HA sequences of two EU swine influenza vaccine strains and five human seasonal influenza vaccine strains (from seasons 2008–2021) were obtained from the publicly available database, Global Initiative on Sharing Avian Influenza Data (GISAID EpiFlu; http://platform.gisaid.org/epi3/; accessed in August 2020).^14^ Strain information regarding commercial use EU swine influenza vaccine was based on literature review (Table 1A).^15^, ^16^ Sequences of the US swine influenza vaccines, namely, FluSure (FS)XP/IA00, FSXP/OK08, and FSPandemic (FSPDM)/CA09, were provided by Zoetis. Experimental COBRA influenza vaccine sequences including swine (SW1 and SW2), human (X3 and X6), and a hybrid swine/human vaccine, P1, were provided by the Center for Vaccine and Immunology (CVI), University of Georgia (UGA). The COBRA SW1 and SW2 HA antigens were designed to be more cross‐protective antigens using HA sequences from swine H1N1 and H1N2 sequences. Human COBRA X3 and X6 were designed using HA sequences from human isolates, while COBRA P1 was derived from both swine and human H1 HA sequences.^17^

**TABLE 1A:** Source of H1 HA sequences used in the analyses

Region	Host	Strain name	Category	Label	Accession no.
EU	Swine	A/swine/Bakum/1832/2000	Commercial	BK00	EU053148
		A/swine/Haseluenne/IDT2617/2003		HL03	GQ161119
US	Swine	A/swine/Iowa/110600/2000	Commercial	FSXP/IA00	Not available
		A/swine/Oklahoma/0726H/2008		FSXP/OK08	
		A/California/04/2009		FSPDM/CA09	EPI_ISL_393964
		COBRA/SW1	Experimental	COBRA/SW1	Not available
		COBRA/SW2		COBRA/SW2	
	Human and swine hybrid	COBRA/P1		COBRA/P1	Not available
	Human	COBRA/X3		COBRA/X3	
		COBRA/X6		COBRA/X6	
Global		A/Brisbane/59/2007	Seasonal	BR07	KF009550
		A/California/07/2009		CA09	CY121680
		A/Michigan/45/2015		MI15	KU933493
		A/Brisbane/02/2018		BR18	EPI1692062
		A/Guangdong‐Maonan/SWL1536/2019		GDMN19	EPI1719956

**TABLE 1B:** H1N1 G4 swIAV strains included in analyses

No.	Strain name	Accession no.
1	A/swine/Heilongjiang/1214/2016	MN416609
2	A/swine/Jilin/21/2016	MN416627
3	A/swine/Shandong/1207/2016	MN416643
4	A/swine/Hebei/0113/2017	MN416596
5	A/swine/Anhui/0202/2018	MN416586
6	A/swine/Beijing/0301/2018	MN416589
7	A/swine/Henan/SN11/2018	MN416620
8	A/swine/Jiangsu/J006/2018	MN416626

#### 2.1.2 G4 and circulating strains

**FIGURE 1:**
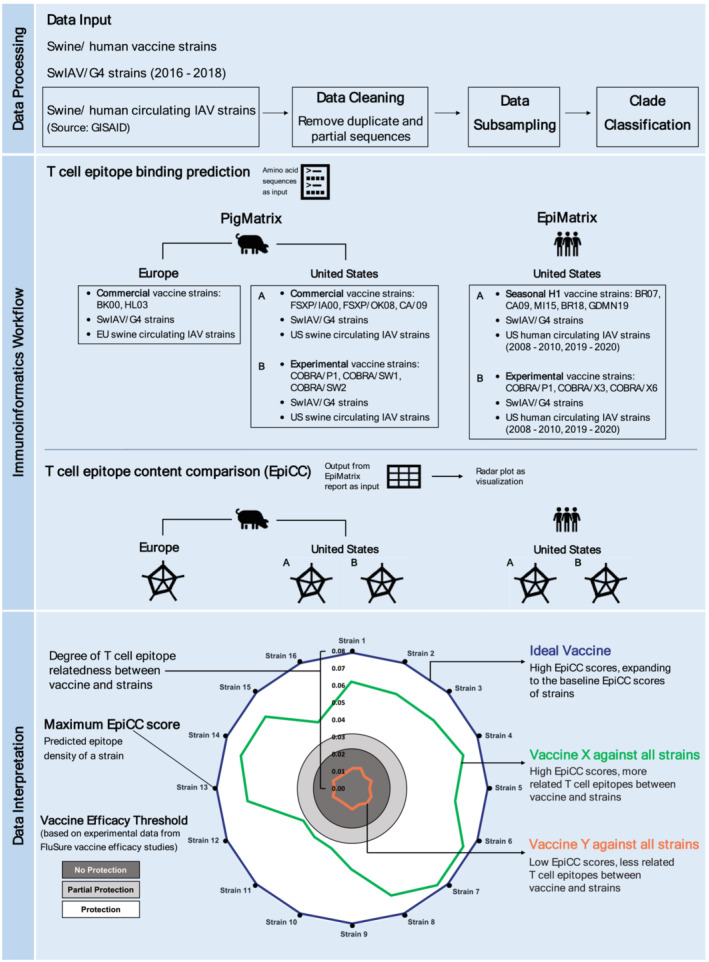
Analysis workflow to quantify the degree of epitope conservation between target vaccines, G4, and circulating influenza A virus (IAV) strains. The key steps include data processing, immunoinformatics workflow, and data interpretation.

Twenty‐nine HA sequences from previously published swine H1N1 G4 genotype strains were used in this study.^6^ Given that the 29 HA G4 strains were derived from a shorter time range (2016–2018) and that there is great similarity (percentage identity in the range of 95.8%–100.0%) between these sequences, eight sequences were randomly selected for analysis in this study (Figure 1 and Table 1B). All available H1 HA sequences comprising swIAV strains circulating in the US (1939–2020) and EU (1939–2018) and human IAV strains circulating in the US (from 2008–2010 and 2019–2020) were retrieved from GISAID.

#### 2.1.3 Data curation

Duplicate and partial sequences containing less than 1400 nucleotides were removed using a publicly available python script.^18^ Phylogenetic analysis was performed following sequence alignment using MUSCLE 3.8.31.^19^ Maximum‐likelihood phylogenetic trees were constructed with RAxML.v8 using the GTR‐GAMMA nucleotide substitution model.^20^ To ensure computational tractability and to preserve representative clades, Phylogenetic Diversity Analyzer (PDA) was used to subsample 150 sequences each from large dataset that consisted of swIAV strains circulating in the US and EU, respectively, as well as 300 sequences from human IAV strains circulating in the US.^21^ The final reduced dataset (supporting information Table S1A–C) was translated into amino acid sequences and combined with selected G4 strains and respective vaccine strains for three sets of analyses (Figure 1): (1) European circulating swine flu virus and G4 strains, (2) US circulating swine flu and G4 strains, and (3) US circulating human influenza virus and G4 strains. To better classify strains according to their respective phylo‐clusters, metadata such as H1 strain clade information was acquired using Swine H1 Clade Classification Tool.^22^

### 2.2 T cell epitope binding prediction

We initiated our analysis by focusing on HA, given its importance as the critical antigen that is most relevant to protective immunity to influenza. Additional antigens were also evaluated (see Section 3.3). After compiling IAV sequences as described in Section 2 and illustrated in Figure 1, the translated HA protein sequences were screened using host‐specific T cell epitope identification algorithms developed by EpiVax. Particularly, PigMatrix epitope prediction tools were used for the swine sequences, and EpiMatrix was used for identification of human epitopes. These prediction tools parse sequences into overlapping 9‐mer frames to define the relative likelihood of binding to a set of prevalent swine leukocyte antigen (SLA) or supertype human leukocyte antigen (HLA) class I and II alleles.^23^, ^24^

More specifically, using a position‐specific scoring matrix, predicted binding scores were computed by PigMatrix for the likelihood of each 9‐mer in the HA antigens of IAV to bind to a panel of prevalent class I (SLA‐1*01:01, 1*04:01, 1*08:01, 1*12:01, 1*13:01, 2*01:01, 2*04:01, 2*05:01, 2*12:01, 3*04:01, 3*05:01, 3*06:01, and 3*07:01) and class II (SLA‐DRB1*01:01, 02:01, 04:01, 04:02, 06:01, 06:02, 07:01, and 10:01) SLA alleles. The SLA allele selection for swine reflects dominant SLA types, each of which has binding pocket preferences that are shared with several other SLA alleles. This approach was based on a previous study reported by Gutierrez et al.^25^, ^26^ Similarly, predicted binding scores for each 9‐mer from human IAV sequences were assigned by EpiMatrix, and the HLA alleles that were used in the prediction model include class I (A*01:01, A*02:01, A*03:02, A*24:02, B*07:02, and B*44:03) and class II (DRB1*01:01, DRB1*03:01, DRB1*04:01, DRB1*07:01, DRB1*08:01, DRB1*09:01, DRB1*11:01, DRB1*13:01, and DRB1*15:01) HLA molecules. These are HLA allele supertypes (alleles sharing common binding preferences) that cover the genetic diversity of more than 95% of human populations globally.^23^, ^27^, ^28^

For each 9‐mer, *i* in each individual allele *a* of a set of MHC alleles A, PigMatrix or EpiMatrix raw scores, *r*, are normalized to *Z*‐scores using the average μ and the standard deviation σ of scores calculated for 100,000 random 9‐mers using the formula below.^23^, ^24^ Nine‐mers with *Z*‐scores greater or equal to 1.64, which comprise the top 5% in the normalized set of scores for each SLA or HLA allele of sequences, are predicted to have significant SLA or HLA binding potential. Higher *Z*‐scores associate with higher MHC binding probability.^26^, ^29^

$$Z{\left(i\right)}_{a}=\frac{\left(r-\mu \right)}{\sigma }$$

### 2.3 Analysis of T cell EpiCC

We applied EpiCC to facilitate the pairwise T cell EpiCC of protein sequences (Figure 1). EpiCC enables large‐scale sequence analysis for conservation of T cell epitopes between swine and human flu vaccines and circulating IAV and G4 strains, focusing only on shared T cell epitopes between the vaccines and the target IAV strains.^26^, ^30^ Once T cell epitope content is defined for each vaccine or strain, the set of conserved T cell epitopes that are shared between two strains can be enumerated.

In mathematical terms, EpiCC assesses the relatedness of T cell epitope, *i*, contained in a protein sequence of vaccine strain *v* and T cell epitope, *j*, contained in a protein sequence of a strain *s* based on respective PigMatrix SLA binding or EpiMatrix HLA binding score. Since cross‐reactive memory T cells can be stimulated by epitopes (*i*, *j*) with identical TCR‐facing residues (TCR*f*) that may have different HLA binding pocket residues, as long as they bind to the same alleles, we searched for potentially cross‐reactive epitopes that shared TCR*f* as follows: Cross‐conserved class I epitopes were defined by identical residues at positions 4, 5, 6, 7, and 8, and class II epitopes were defined by identical residues at positions 2, 3, 5, 7, and 8. The score of cross‐conserved T cell epitope shared between two strains *s* and *v* was calculated using predicted binding probabilities as follows:

$$S{\left(i,j\right)}_{a}=p{\left(i\right)}_{a}\cdot p{\left(j\right)}_{a}$$

To normalize shared EpiCC score, the sum of shared epitope scores of each *i*, *j*, was normalized by the total number of compared pairs, *p*, and by the number of MHC alleles in A. This is to account for different epitope densities and for comparison of values of E determined using different numbers of MHC alleles. Therefore, the normalized shared EpiCC score, (termed as EpiCC score), can be computed by applying the following equation:

$$E{\left(\text{shared}\right)}_{A}=\frac{1}{|p|\cdot |A|}\sum_{i\in s,j\in v}\sum_{a\in A}S{\left(i,j\right)}_{a}$$

Maximum EpiCC scores were calculated. These scores were derived from shared EpiCC scores computed from the comparison of any sequence to itself. The greater the maximum EpiCC score, the greater the total epitope content of the sequence. Since no sequence can be better matched to another sequence than itself, the maximum value for any comparison between any target sequence and a comparison sequence is always less than or equivalent to their maximum EpiCC scores. Both class I and class II EpiCC analyses were combined by summing class I and class II EpiCC scores (termed as total EpiCC score) for each vaccine‐to‐strain comparison.

When the shared T cell epitope content of a strain of influenza is highly related or “covered” by a given influenza vaccine sequence, the vaccine‐to‐strain's EpiCC score approaches the circulating strain's maximum EpiCC score (it approaches the maximum if nearly all the epitopes are identical, as defined above). To determine vaccine‐to‐strain EpiCC scores coverage, each vaccine‐to‐strain comparison was divided by that strain's maximum EpiCC score and expressed as a percentage. The greater the T cell epitope coverage (percentage), the better the vaccine matches or covers the T cell epitope content of the circulating strain sequences (Figure 1).

### 2.4 EpiCC scores and EU vaccine efficacy estimation

To identify a threshold of protective efficacy of existing swine vaccines against circulating field strains, we extrapolated from available data, using an approach similar to one that we have already published.^26^ For HA sequences, we calculated the EpiCC scores for three H1N1 EU commercial vaccines and three experimental monovalent vaccines against EU circulating swine IAV strains from the same period. Once we had obtained the scores, we identified the minimum EpiCC scores that correlated with protective endpoint results in four published studies that used the commercial and/or experimental vaccine strains. Vaccines were protective if they significantly reduced lung virus titers. The EpiCC score protective threshold was defined as the lowest EpiCC for at which the vaccine strain was shown to be protective. This is the main criterion for evaluating protection in the EU.^16^ Scoring was performed independently of and prior to obtaining information about the outcomes of the vaccination and challenge studies.

### 2.5 Statistical analysis

A non‐parametric Wilcoxon signed‐rank test was used to compare T cell epitope coverage of different groups of vaccines (swine and human) analyzed against G4. *P*‐values (*p*) less than 0.05 were deemed significant. The analysis was performed using the rstatix package in R version 4.0.3.^31^

### 2.6 Analysis on other IAV antigens

To determine whether other viral antigens might contribute to the protective efficacy of vaccines and circulating strains, we extended and applied similar workflow as described above to additional nine antigens other than HA. The nine antigens included neuraminidase (NA), polymerase basics (PB1 and PB2), polymerase acidic (PA), nucleoprotein (NP), matrix proteins (M1 and M2) and non‐structural proteins (NS1 and NS2). The purpose of this analysis was to compare and assess the degree of T cell epitope relatedness between whole‐killed vaccines strains, IAV circulating strains, and G4 of non‐HA antigens. Available whole proteome sequences of EU swine flu vaccines, human seasonal influenza vaccine strains (Table 1A), and the eight G4 sequences (Table 1B) were obtained from GISAID (accessed in December 2021). Sixteen clade‐specific swine H1 IAV strains circulating in EU and five human H1 IAV strains circulating in the US were randomly selected from the previously defined dataset for analysis (supporting information Table S1A,C).

## 3 RESULTS

We set out to evaluate the potential for cross‐conserved T cell immune responses to protect against G4 in swine and human populations using immunoinformatics methods. The results of this analysis are divided into two parts due to species‐specific MHC binding preferences (swine and human) and the species‐specific circulation patterns of influenza strains. The first part of the analysis focused on protection by swine vaccines against G4 in swine, and the second part focused on predicting protection against G4 influenza in case of spillover into human hosts.

For the swine IAV analysis, we evaluated two commercial H1N1 swine influenza strains used in the EU and three US commercial H1N1 vaccine strains against strains from their respective regions of the world. We also evaluated the experimental (swine) COBRA vaccines that were previously studied for cross‐reactive antibodies, for T cell epitope conservation against G4 using swine epitope prediction tools (PigMatrix and EpiCC). In the second section, we evaluated human T cell epitope content relatedness of five seasonal (human) H1N1 IAV vaccine strains and three experimental (human COBRA) IAV vaccine strains in the US human population to determine their potential to control G4 in the event of G4 spillover into the US human population, using EpiMatrix and EpiCC.

**FIGURE 2:**
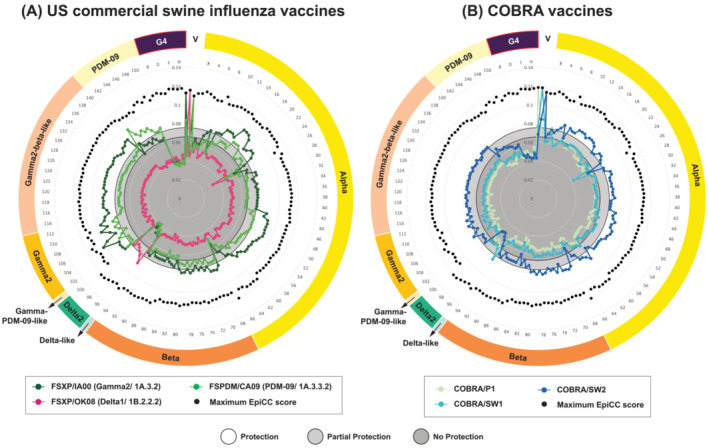
This radar plot of the EpiCC analysis enables the comparison of T cell epitope relatedness of the US swine influenza vaccines (A: Commercial; B: COBRA) to circulating US swIAV and G4 strains. EpiCC scores are plotted in a radial fashion in order of chronological time while also grouping flu variants into strain families, and color‐coded lines represent each of the vaccine strains (V) compared with each of the circulating strains. Each of the vaccine strain labels is shown in legend. The ring surrounding the radar plot identifies the swIAV sequences metadata using the US clade naming system, which includes alpha, beta, delta, gamma, and pandemic 2009 lineage (PDM‐09). The two shaded gray circles near the center of the radius define vaccine efficacy thresholds as reported in Gutiérrez et al. 2017 [26]. Refer to Figure 1—Data interpretation for more details. As indicated in both A and B, the G4 strain has few cross‐conserved T cell epitopes with vaccine strains (all vaccines' EpiCC scores fall below the protective thresholds).

**FIGURE 3:**
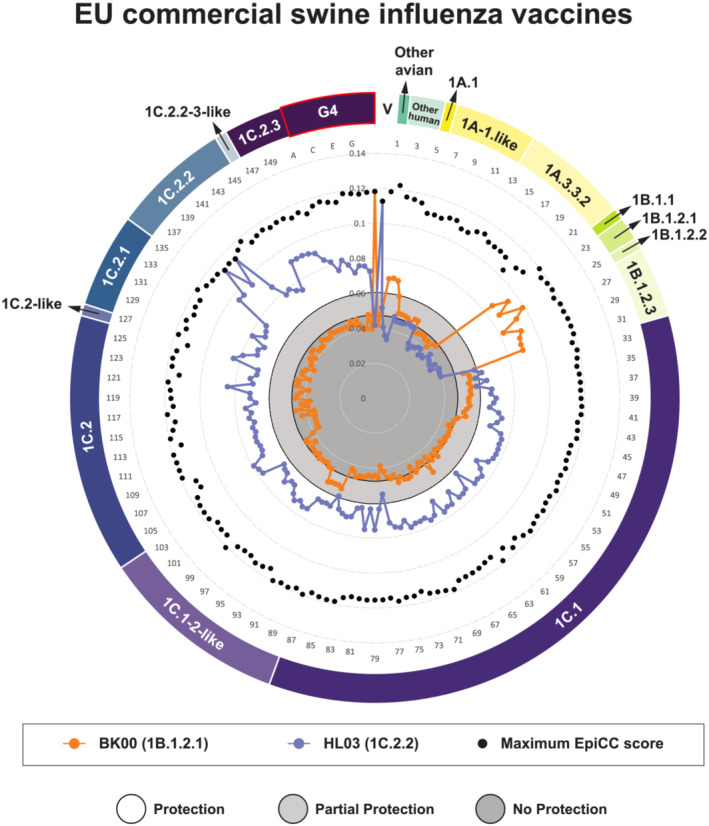
Comparison of total (class I and class II) shared T cell epitope relatedness of European swine influenza vaccines against EU circulating swIAV and G4 strains as measured using the EpiCC algorithm. Representative individual strain sequences are numbered, and their clade designation is labeled in the outer ring surrounding the radar plot. The letter “V” designates the EpiCC scores for the European vaccine strains. The experimentally defined protection thresholds for vaccine efficacy are shown by the shaded gray circles. Note that the observed EpiCC scores indicate that the European vaccine strain, HL03, may provide protective immunity against G4.

**FIGURE 4:**
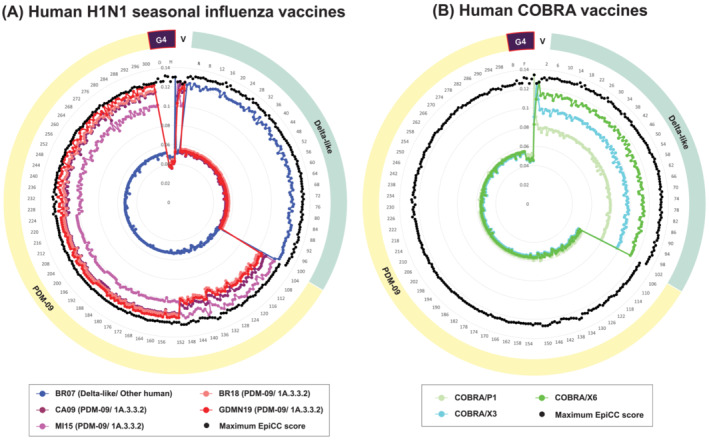
EpiCC analysis of human influenza vaccines against US circulating human IAV and G4 strains (A: human H1N1 seasonal influenza vaccines; B: Human COBRA influenza vaccines). The outer ring surrounding the radar plot shows sequence metadata. The symbol “V” represents vaccine strains, and each vaccine strain is color‐coded as indicated in the legend. A protective vaccine efficacy T cell epitope conservation (EpiCC) threshold for human influenza vaccine efficacy has not been defined, as most influenza vaccines generate cross‐reactive antibody. Note that EpiCC scores for G4 are extremely low compared to scores for other strains.

As described above, T cell epitope content relatedness was defined as the density of shared T cell epitope content between the vaccine of interest and targeted strain (EpiCC score). A percent coverage was used to normalize score and permit comparison to established protective thresholds. Figure 1 illustrates how radar plots are constructed; the results of the swine and human vaccine‐to‐strain analyses are shown in separate radar plots (Figures 2, 3, 4), combining MHC class I and II comparisons (SLA for swine; HLA for human).

In the sections below, we described the results of the EpiCC analysis for HA, as it is the principal target of protective immune responses in influenza infection and is the most variable sequence in the pathogen.^32^ Changes to vaccine composition and changes in vaccine efficacy are primarily due to drift and shift in the sequence of HA antigen. In Section 3.3, we provided results for additional influenza proteins, to allow for a more comprehensive understanding of their potential to contribute to changes in T cell epitope content.

### 3.1 Swine vaccine‐to‐strain EpiCC analysis results

#### 3.1.1 EpiCC analysis of US vaccines and strains

EpiCC scores comparing the T cell epitope content of the HA of three commercial swine H1N1 vaccines used in the US (FSXP/IA00, FSXP/OK08 and FSPDM/CA09) to 150 swIAV strains circulating in the US from 1939 to 2020 and eight G4 sequences were calculated (Figure 1A). Overall, for these swine vaccine‐to‐strain comparisons tended to be clade‐specific. Higher total EpiCC scores (greater cross‐conservation) were observed for FSXP/IA00 when it was compared with US swIAV circulating strains. The average of vaccine T cell epitope coverage was 69.4% for this comparison. In contrast, the average T cell epitope coverage by FSXP/OK08 and FSPDM/CA09 of US swIAV strains was lower, at 43.2% and 59.9%, respectively. FSXP/IA00 had high T cell epitope relatedness to swIAV circulating strains from eight clades (Alpha, Beta, Gamma‐PDM‐09‐like, Gamma2, Gamma2‐beta‐like, and PDM‐09) although Delta‐like and Delta2 EpiCC scores were lower. FSXP/OK08 (HA from H1N2) only exhibited T cell epitope content shared with swIAV Delta‐like and Delta2 strains. The third vaccine strain, FSPDM/CA09, which has been in use since 2009, demonstrated high T cell epitope relatedness to swIAV strains from PDM‐09, Gamma‐PDM‐09‐like, and Gamma2 lineages.

EpiCC scores that were previously defined using experimental data available for FSXP/IA00 as fully protective or partially protective thresholds in Gutiérrez et al. (2017) (in the absence of cross‐reactive antibodies)^26^ were extrapolated and applied to the swine vaccine‐to‐strain EpiCC analysis. A total EpiCC score of at least 0.076 was used to define complete protection; a score between 0.065 and 0.076 was used to define partial protection (light and dark gray circles, respectively, in Figure 2). Based on these protective thresholds, FSXP/IA00 was predicted to confer protection and partial protection against all swine influenza strains except Delta2 and G4. A summary of US swine influenza vaccines EpiCC scores and vaccine coverage can be found in Table 2. Statistical analysis was also performed to evaluate the potential of protection against G4, and FSXP/IA00 was predicted to have lower T cell epitope coverage and was therefore considered not likely to provide protection (*p* < 0.05; supporting information Figure S1). Other US commercial swine influenza vaccines had low T cell epitope coverage as well and were predicted to confer no protection.

**TABLE 2:** Average EpiCC score and vaccine T cell epitope coverage of US swine influenza vaccine strains compared with US circulating swIAV and G4 strains

Vaccine			FSXP/IA00	FSXP/OK08	FSPDM/CA09	COBRA/SW1	COBRA/SW2	COBRA/P1^a^
Type			Commercial			Experimental		
US circulating IAV strains (*n =* 150)	Average EpiCC score (×10^−2^)	Class I	3.49	1.98	2.98	2.48	3.27	2.35
		Class II	4.35	2.90	3.78	3.54	4.26	3.26
		Total	7.84	4.88	6.76	6.02	7.53	5.61
	Average maximum EpiCC score (sd)^b^		11.29 (0.25)					
	Vaccine coverage (%)^c^		69.4	43.2	59.9	53.3	66.7	49.7
	Predicted vaccine efficacy^¶^		Protective	Not protective	Partial protective	Not protective	Protective	Not protective
G4(*n =* 8)	Average EpiCC score (×10^−2^)	Class I	1.69	1.69	1.69	1.77	1.97	2.06
		Class II	2.20	2.50	2.12	2.81	2.59	2.93
		Total	3.89	4.19	3.81	4.58	4.56	4.99
	Average maximum EpiCC score (sd)^b^		11.66 (0.15)					
	Vaccine coverage (%)^c^		33.4	35.9	32.7	39.3	39.1	42.8
	Predicted vaccine efficacy^d^		Not protective	Not protective	Not protective	Not protective	Not protective	Not protective

^a^ COBRA/P1 is an experimental vaccine that derived from both swine and human HA sequences (termed as ‘hybrid’ in this study).

^b^ Average maximum EpiCC score (and standard deviation) of full‐length swIAV strains, expressed in ×10^−2^.

^c^ Vaccine T cell epitope coverage range: >65%: protective, 56.6%–65%: partial protective, and <56.6%: non‐protective.

^d^ Vaccine efficacy threshold defined in Gutiérrez et al. (2017) using experimental data that served as a proxy for this analysis.

COBRA vaccines are computationally optimized antigens designed to provide broad antibody epitope coverage against a wide range of variable sequences, but their T cell epitope content is not currently optimized. We considered the T cell epitope coverage of three experimental COBRA vaccines (COBRA/P1, COBRA/SW1, and COBRA/SW2)^17^ for the set of US swIAV strains used in this study. COBRA/SW2 showed the highest T cell epitope relatedness to US field strains, with vaccine T cell epitope coverage of 66.7% (Table 2). COBRA/SW1 and COBRA/P1 had lower vaccine T cell epitope coverage of 53.3% and 49.7%, respectively. Again, the vaccines were clade‐specific: COBRA/SW2 had greater total shared T cell epitope relatedness to field strains from Alpha, Beta, Gamma2, Gamma2‐beta‐like, and PDM‐09 clades (Figure 2B). It also had less T cell epitope relatedness to Delta‐like and Delta2 field strains. In contrast, COBRA/SW1 was predicted to confer protection only against Delta‐like swIAV field strains, while COBRA/P1 was predicted to confer only partial protection against Delta‐like field strains. These results suggest that similar patterns of T cell epitope relatedness are observed among circulating strains from the same lineage (classical swine influenza versus human seasonal influenza).

#### 3.1.2 EpiCC analysis of EU vaccines and strains

For EU strains, we established thresholds for full and partial protection using retrospective data. The commercial and experimental inactivated virus vaccine strains used in challenge studies were considered protective in our analysis if lung virus titers were significantly lower than the challenge control group and pigs showed no and/or mild clinical signs (low mean temperature or low scores of respiratory diseases) compared with unvaccinated pigs. Partial protection was defined as a significant reduction of lung lesions coupled with a non‐significant reduction of lung virus titer when compared with unvaccinated controls.

To define the protective thresholds, we evaluated four published vaccine efficacy studies^33^, ^34^, ^35^, ^36^ which used four different H1N1 challenge strains. Table 3 lists all the vaccine strains that conferred protection or partial protection against specific challenge strains and the relevant lung titer data. In six of the eight evaluations, protection was demonstrated by the absence of virus, or by significantly lower lung virus titers than the control group. A shared T cell epitope content EpiCC score was calculated for each vaccine‐to‐strain comparison (Table 3).

**TABLE 3:** Commercial and experimental vaccination and H1N1 challenge studies in EU

No.	Vaccine		H1N1 challenge virus^a^	Mean HI antibody titers prior to challenge		Virus titer in lungs	Clinical outcome	EpiCC score (×10^−2^)			Ref.
	Product name	H1N1 strain		Against homologous vaccine strain	Against challenge virus			Class I	Class II	Total	
1	Gripovac®	NJ76	BE/1/83	80–320^b^	40‐160^b^	Negative	Protection	3.50	2.54	6.04^c^	[33]
2	Gripovac®	NJ76	GT/112/07	86	5	Significantly lower (only in the left lung half)	Partial protection	2.73	2.01	4.74^d^	[34]
	Suvaxyn® Flu	NL80		80	10	Significantly lower	Protection	4.02	2.87	6.89	
3	Experimental	NJ76	BE/1/98	305	16	No significant difference	Protection	2.73	2.08	4.81	[35]
		BE83		235	91	Significantly lower	Protection	4.26	3.33	7.60	
		BE98		197	197	Significantly lower	Protection	6.44	5.29	11.73	
	Gripovac®	NJ76		610	197	Significantly lower	Protection	2.73	2.08	4.81	
4	Respiporc® Flu3	HL03	PD/15/1981	> 256	Not detected	Significantly lower	Protection but less effective	3.80	2.84	6.64	[36]

*Note*: This analysis focused on the H1N1 component of previously available swine influenza vaccines in EU or experimental swIAV strains. The primary criterion for assessing vaccine protection in EU vaccine challenge studies was measurement of lung virus titers [14, 15].

Abbreviation: EpiCC, Epitope Content Comparison.

^a^ All H1N1 challenge viruses were all swine strains, with abbreviations BE: Belgium; GT: Gent; PD: Potsdam.

^b^ Original data reported HI antibody titers prior to challenge against homologous vaccine strain/challenge virus as range instead of mean.

^c^ Minimum EpiCC score that predicted to confer protection.

^d^ Minimum EpiCC score that predicted to confer partial protection.

Based on these studies, the lowest total EpiCC score that was associated with protective efficacy was 0.0604, defined by comparing T cell epitopes from the swine/Belgium/1/83 challenge strain with the first generation of H1N1 European vaccine strain (NJ76). This threshold is represented by the white area in Figure 3. Based on this study and previous studies, swIAV strains that have total EpiCC scores above the threshold are likely to be protective. Similarly, a total EpiCC score of 0.0474 was associated with partial protective efficacy, as defined for the T cell epitope comparison between NJ76 and challenge strain GT/112/07. For this analysis, therefore, we defined total EpiCC score between 0.0474 and 0.0604 as partially protective.

#### 3.1.3 Protective efficacy of EU swine influenza vaccines against circulating and G4

Having established estimated vaccine efficacy thresholds for EU swine influenza vaccines, we could evaluate whether the T cell epitope content of additional swIAV vaccine strains that are commonly used in the EU commercial settings (BK00 and HL03) might protect against 150 swIAV strains circulating in the EU and eight emergent G4 sequences (Figure 3).

HL03 had the highest T cell epitope coverage (vaccine‐to‐strain EpiCC scores) for circulating swIAV strains in the EU and was predicted to provide protection against 76% of the swIAV strains (total EpiCC scores greater than 0.0604). The BK00 strain had lower T cell epitope relatedness against EU swIAV circulating strains (only 8.7% of EU swIAV circulating strains had total EpiCC score of at least 0.0604). EpiCC comparisons showed that T cell epitope cross‐conservation was lineage specific: HL03's EpiCC score suggested that it may only confer protection against 1C lineages strains. In contrast, vaccine strain BK00's EpiCC score suggests that it may confer protection to field strains related to 1B and other avian or human lineages but not 1C lineages.

Notably, the HL03 vaccine strain had total EpiCC scores that exceeded the defined protective threshold for emergent G4. The average HL03 vaccine strain T cell epitope coverage (slate blue line in Figure 3) for the G4 HA sequences was 65.7%, which is above the protective threshold (Table 4). This observation suggests that existing European swine vaccines may have a protective effect against emergent virus G4.

**TABLE 4:** Average EpiCC score and vaccine T cell epitope coverage of EU swine influenza vaccine strains compared with EU circulating and G4 strains

Vaccine			BK00	HL03
Type			Commercial	
EU circulating IAV strains (*n* = 150)	Average EpiCC Score (×10^−2^)	Class I	2.07	2.93
		Class II	2.86	3.73
		Total	4.93	6.66
	Average maximum EpiCC score (sd)^a^		11.55 (0.28)	
	Vaccine coverage (%)^b^		42.7	57.7
	Predicted vaccine efficacy^c^		Partially protective	Likely protective
G4(*n* = 8)	Average EpiCC Score (×10^−2^)	Class I	1.80	3.46
		Class II	2.52	4.20
		Total	4.32	7.66
	Average maximum EpiCC score (sd)^a^		11.66 (0.15)	
	Vaccine coverage (%)^b^		37.0	65.7
	Predicted vaccine efficacy^c^		Not protective	Likely protective

Abbreviation: EpiCC, Epitope Content Comparison.

^a^ Average maximum EpiCC score (and standard deviation) of full‐length swIAV strains, expressed in ×10^−2^.

^b^ Vaccine T cell epitope coverage range for EU swine influenza vaccines: >51.8%: protective, 40.6%–51.8%: partial protective, <40.6%: non‐protective.

^c^ Vaccine efficacy threshold as defined in Table 3 that served as a proxy for this analysis.

### 3.2 Human vaccine‐to‐strain EpiCC analysis results

To assess whether existing human vaccines would provide protection against potential spillover of G4 into US human population, we evaluated the T cell epitope relatedness of five commercial human H1N1 seasonal influenza vaccine strains to G4 using EpiCC (Figure 4 and Table 5).

**TABLE 5:** Average EpiCC score and vaccine T cell epitope coverage of US human influenza vaccine strains compared with US human circulating IAV and G4 strains

Vaccine			BR07	CA09	MI15	BR18	GDMN19	COBRA/X3	COBRA/X6	COBRA/P1^a^
Type			Seasonal H1N1					Experimental		
US human circulating IAV strains (*n* = 300)	Average EpiCC score (×10^−2^)	Class I	3.57	4.27	4.56	4.69	4.74	3.20	3.57	3.03
		Class II	3.95	4.56	4.72	4.64	4.77	3.63	3.88	3.34
		Total	7.52	8.83	9.28	9.33	9.51	6.83	7.45	6.37
	Average maximum EpiCC score (sd)^b^		12.76							
	Vaccine coverage (%)		58.9	69.2	72.7	73.1	74.5	53.5	58.4	49.9
G4 (*n* = 8)	Average EpiCC score (×10^−2^)	Class I	2.02	2.08	2.00	2.06	2.00	2.06	2.12	2.41
		Class II	2.66	1.96	1.92	2.12	1.96	2.50	2.55	2.71
		Total	4.68	4.04	3.92	4.18	3.96	4.56	4.67	5.12
	Average maximum EpiCC score (sd)^b^		12.84							
	Vaccine coverage (%)		36.4	31.5	30.5	32.6	30.8	35.5	36.4	39.9

Abbreviations: IAV, influenza A virus; EpiCC, Epitope Content Comparison.

^a^ COBRA/P1 is an experimental vaccine that derived from both swine and human HA sequences (termed as “hybrid” in this study).

^b^ Average maximum EpiCC score (and standard deviation) of full‐length human IAV strains, expressed in ×10^−2^.

One seasonal vaccine strain, BR07, had a distinctive pattern with high T cell epitope content relatedness when compared with Delta‐like human IAV circulating strains such as strains that were circulating prior to the 2009 pandemic (Figure 4A). As expected, CA09, which was introduced in the 2009 pandemic, demonstrates high T cell epitope relatedness when compared with circulating strains of PDM‐09 (Figure 4A). Newer H1N1 vaccine strains that were introduced after the CA09 pandemic also had high EpiCC scores when compared with PDM‐09 circulating strains (approximately 70.0% T cell epitope coverage on average). T cell epitope coverage for human vaccine‐to‐G4‐strains comparison was much lower, at 32.4% on average (Table 5).

Unlike the vaccine efficacy studies performed in swine where challenge studies data such as lung virus titers and lung lesion reduction are accessible, human vaccine efficacy is determined via clinical trials.^37^ Human vaccine efficacy estimates vary among published efficacy studies,^37^ and hence, defining an EpiCC protective threshold is not straightforward. Since the protective thresholds were not defined for human vaccines, we used an average vaccine‐to‐strain EpiCC scores coverage of 38.5% (represented by the dotted line in supporting information Figure S1) to evaluate the T cell epitope coverage for human vaccines (supporting information Figure S1). The human seasonal influenza vaccines (CA09, MI15, BR18, and GDMN19) showed T cell epitope coverage below the mean and had no significant T cell epitope relatedness to G4 (*p* < 0.05) when compared with G4.

Three novel subunit influenza vaccines (COBRA HA vaccines) that were designed to generate cross‐protective B cell epitopes^17^ were also compared with both G4, and circulating human IAV strains, to estimate whether they might also generate protective T memory response against emerging IAV strains. The experimental COBRA vaccine strains only showed T cell epitope content relatedness to pre‐PDM strains of human IAV (Figure 4B). COBRA vaccine T cell epitopes were poorly conserved with G4, with an average of only 37.3% T cell epitope coverage, as was observed for H1N1 seasonal influenza vaccines (Table 5).

### 3.3 T cell epitope conservation among antigens other than HA

Although HA has been the focus of antigenic studies for most influenza vaccines, there is evidence that internal genes such as PB2 and NP may be associated with milder clinical signs and decreased virus shedding.^38^ Therefore, we examined the degree of T cell epitope conservation in other viral antigens. Even though conventional inactivated virus vaccines are manufactured as high‐producing reassortants containing the HA and NA of target strains and internal genes from the master strain (A/Puerto Rico/8/1934),^39^ comparing the T cell epitope content of non‐HA proteins of G4 and circulating H1N1 viruses may shed light on the potential for seasonal influenza virus infection to protect against G4.

All publicly available non‐HA protein sequence data were retrieved for seasonal vaccine strains, G4 and circulating IAV strains. Given that there is no complete proteome sequence for the US swine influenza vaccine strains, this full proteome analysis was only performed for EU swine and US human IAV strains. There were variations in terms of the degree of T cell epitope conservation among viral antigens (supporting information Figure S2A,B and Table S2A,B). For the EU swIAV internal protein analysis, T cell epitopes were found to be conserved between G4 and EU field strains for the PB2, PB1, PA, and NP proteins. T cell epitope conservation was particularly high between HL03 and G4 for the M1 protein. Lower T cell epitope conservation was observed between EU vaccine strains and G4 for the NA protein and other internal antigens such as M2, NS1, and NS2 (average vaccine coverage less than 65%, supporting information Figure S2A and Table S2A).

As compared with EU swine vaccine strains, the internal antigens of the human vaccine strains for the US, particularly CA09 and MI15, had higher T cell epitope conservation with human circulating strains and G4 (supporting information Figure S2B and Table S2B). Internal antigens PB2, PB1, PA, NP, M1, and M2 had total EpiCC scores of at least 0.097, that is, at least 85% T cell epitope coverage (supporting information Figure S2B and Table S2B). In contrast, internal proteins from the vaccine strain BR07 had lower T cell epitope conservation with G4, with T cell epitope coverage ranging between 12.1% and 75.7%. This suggests that the T cell epitopes from the internal antigens of CA09 and MI15 (H1N1/pdm09 lineage) are more highly conserved with T cell epitopes from the internal antigens of G4.

## 4 DISCUSSION

Vaccination remains the most effective public health intervention for combatting influenza infections in both swine and humans. However, the influenza virus is constantly undergoing drift and shift events, making it difficult for some vaccine strains to provide adequate protection. This is particularly worrisome when influenza viruses with reported pandemic potential, such as G4, begin to emerge in the established host population or are shown to possess critical adaptations that allow infection and possible transmission to a new host. While antibodies are usually considered to be the major correlate of protection following influenza vaccination, influenza vaccines containing highly cross‐conserved T cell epitopes have also been shown to reduce morbidity and limit spread in the absence of antibodies, even when there is a mismatch between vaccines and emergent strains.^10^, ^11^, ^12^

To assess the potential epidemic risk posed by the emergent G4 in swine and human populations that are naïve to this virus, we compared the HA antigen T cell epitopes contained in three US commercial swIAV vaccine strains, two commercial‐use EU swIAV vaccine strains, five seasonal H1N1 human IAV vaccine strains, and five experimental COBRA vaccines (two strains designed for each swine and human host and one hybrid strains) to the T cell epitopes contained in emergent G4 HA and to circulating IAV strains from each respective region and host.

Approaches for influenza vaccination differ between swine and humans, while human vaccines rely on the World Health Organization (WHO) to make annual vaccine strain recommendations and no standardized guidelines have been established for swine vaccine strains and dosages.^16^ Moreover, the strains of influenza used in vaccines for swine differ in Europe and the US due to differences in the requirements for vaccine approval by regulatory agencies. Further variation in vaccine strains may occur, as some US‐based pork producers have been applying for an exemption to the United States Department of Agriculture (USDA) rules to use “autologous” influenza strains.^16^

Current commercial swine influenza vaccines used in the US are polyvalent and contain vaccines targeting distinct circulating H1 and/or H3 strains. For this comparison, we examined the H1 components of the vaccines, consisting of γ‐cluster and δ‐like cluster H1N1 vaccine strains, and δ1‐cluster H1N2 vaccine strain. We determined that neither US commercial nor experimental swine influenza COBRA vaccines were predicted to have high T cell epitope relatedness against G4. The average T cell epitope coverage for G4 was lower than the threshold established for protective T cell activity in previously published studies. This analysis suggests that the US swine population may be susceptible to the emergent G4, even if vaccinated with current commercial vaccines.

The commercial swIAV vaccine strains used in Europe are slightly older, containing H1avN1 (Europe‐avian‐like lineage), H1huN2 (human‐like lineage), and H3N2 subtypes, which are more related to G4. Using published vaccine efficacy data from Europe, we defined putative cross‐protective thresholds for the EU influenza vaccines using a similar approach established by Gutierrez et al.^26^ Lung virus titers are used as the primary measure for defining protective thresholds for Europe swine influenza vaccines rather than reduction in lung lesions. The criteria used in this analysis differed slightly from Gutierrez et al., as lung virus titers data were not available for all challenge strains in their study.^26^ EpiCC analysis demonstrated that vaccine strain HL03 had the highest EpiCC scores against G4, and the potential protection predicted for HL03 was significantly different when compared with other vaccines. G4 contains EA surface proteins (HA and NA).^6^ The EpiCC analysis suggests that only one vaccine strain, HL03, from EA lineage (1C clade) may protect the European swine population against the emergent virus.

While we confirmed that there was high T cell epitope relatedness between seasonal human influenza vaccines with circulating human IAV strains (as expected), there was very low T cell epitope relatedness between human influenza vaccine HA and G4 HA. This suggests that vaccination with seasonal influenza vaccine HA antigen would not induce cross‐protective T cell memory against G4. We also evaluated novel influenza vaccines, known as COBRAs, and found low conservation of the HA antigen T cell epitopes with G4 HA T cell epitopes.

In addition to analyzing the HA protein of swine and human influenza for T cell epitope conservation with the HA of G4, we conducted a more comprehensive protein analysis to compare G4 conservation with other different viral antigens. Phylogenetic analysis and genotype characterization have determined that G4 carries HA and NA from the EA IAV lineage and a mix of internal genes from H1N1/pdm09 and TR lineages.^6^ We hypothesized that vaccine strains that carried H1N1/pdm09 internal genes might have high T cell epitope relatedness to internal antigens of G4. Indeed, we found that internal antigens of human vaccine strains CA09 and MI15 that are from H1N1/pdm09 lineage (all internal antigens except NS antigen) have greater T cell epitope conservation to G4. Even though the HA of G4 is divergent, cross‐conservation of internal protein epitopes between current circulating influenza strains and emergent G4 virus may provide some cross‐protective T cell response to swine and humans.

In conclusion, we estimated the risk of pandemic emergence of the G4 lineage by comparing the T cell epitope content of G4 strains and determining whether the T cell epitope profile matches circulating strains in both human and swine using immunoinformatics approaches. Poor T cell epitope cross‐conservation between G4 and human influenza vaccines may indicate that there is a greater spillover risk to the human population than existed when pH1N1 emerged in 2009. Steps should be taken to prepare for the potential spread of G4 strains. In the absence of G4 vaccines, it may be useful to test available European swine influenza vaccines (HL03) for efficacy against G4. This study also suggests that the emergent G4 may be a greater threat to the US pork industry than to the EU industry, due to the lack of commercial vaccines that could provide cross‐protective immunity to G4. Improving vaccination systems by updating vaccine strains used in pork farms and transitioning to include G4 or EA lineage should be prioritized.

There are limitations in this study that could be addressed in future research. First, while the data subsampling strategy is applied to deal with a large sequence dataset and to avoid overrepresentation of data for certain years or geographical areas, having more data subsampling replicates would better ensure results consistency. Second, the putative cross‐protective thresholds determined for the EU influenza vaccines were based on commercial NJ76 vaccine strains and applied for BK00 and HL03 analyses. While protection thresholds for different vaccine strains may vary, having more experimental data available for BK00 or HL03 would help refine the current thresholds used.

As previously mentioned, T cell epitope conservation between circulating virus strains and seasonal vaccines may contribute to the efficacy of existing (human and swine) influenza vaccines. While this study does not absolutely confirm the relevance of the EpiCC tool for the prediction of human and swine influenza vaccine efficacy, a relationship between EpiCC scores and vaccine efficacy is observed and could be used to establish a threshold for vaccine efficacy in the context of European vaccine strains. In a separate study, EpiCC correctly predicted the efficacy of a novel porcine circovirus type 2 (PCV2) viral vaccine against circulating strains of PCV2 in swine.^40^, ^41^ That prospective study and this retrospective analysis of G4 influenza serve to illustrate the utility of EpiCC analysis for additional prospective studies of existing vaccine strains against emergent strains.

## AUTHOR CONTRIBUTIONS

Swan Tan: conceptualization, formal analysis, methodology, data visualization, writing‐original draft, and writing‐review and editing; Lenny Moise: conceptualization, methodology, writing‐review and editing, and supervision; Douglas S. Pearce: resources and writing‐review and editing; Constantinos S. Kyriakis: writing‐review and editing; Andres H. Gutiérrez: methodology and writing‐review and editing; Ted M. Ross: resources, writing‐review and editing; Justin Bahl: funding acquisition, writing‐review and editing, and supervision; Anne S. De Groot: conceptualization, methodology, writing‐review and editing, and supervision.

## CONFLICT OF INTEREST

Lenny Moise, Andres H. Gutiérrez, and Anne S. De Groot acknowledge that there is a potential conflict of interest related to their relationship with EpiVax and attest that the work contained in this research is free of any bias that might be associated with the commercial goals of the company.

### PEER REVIEW

The peer review history for this article is available at https://publons.com/publon/10.1111/irv.13058.

## Supporting information


**Figure S1.** A vaccine‐to‐vaccine comparison of T cell epitope coverage for each of the vaccine strains against G4. Vaccine strains are color‐coded and arranged in order following host and respective region, year, and vaccine type. The Y‐axis shows vaccine T cell epitope coverage, shown as a percentage of the maximum score. The horizontal dotted line represents mean of T cell epitope coverage for the strains evaluated in this report. Shaded gray areas the threshold for predicted vaccine efficacy for the US (refer to Table 2 footnote) and EU (Table 4 footnote). Only HL03 is predicted to be protective against G4 in this analysis. Statistically significant p‐values are labeled for each comparison.
**Figure S2.** Whole proteome EpiCC analysis of EU swine influenza vaccines (A) and human influenza vaccines (B) against circulating IAV strains in respective regions and G4 strains. Circulating IAV strains were arranged according to clade and year. Red boxes showed H1N1 G4 strains.

**Table S1** A. H1 HA sequences of EU swIAV vaccine strains, swIAV strains circulating in the EU (from 1939–2018) and swIAV G4 virus strains that are included in the analysis.

**Table S2** A. Average EpiCC score, and vaccine T cell epitope coverage of EU swine influenza vaccine strains compared with EU circulating and G4 strains for the whole proteome analysis.

## Data Availability

All sequence data, except US swine influenza vaccine strains (FluSure) and experimental COBRA influenza vaccine sequences, were derived from public resources available at GISAID. Elbe, S. and Buckland‐Merrett, G. (2017) Data, disease, and diplomacy: GISAID's innovative contribution to global health. Global Challenges, 1:33‐46. doi: 10.1002/gch2.1018 PMCID: 31565258 (http://platform.gisaid.org/epi3/).
