# Supplementary material for: H1N1 G4 swine influenza T cell epitope analysis in swine and human vaccines and circulating strains uncovers potential risk to swine and humans
Source: Influenza Other Respir Viruses. 2022 Oct 25;17(1):e13058. doi: 10.1111/irv.13058 (PMC9835423; doi:10.1111/irv.13058)
Supplement: Supplementary file 1 — Figure S1. A vaccine‐to‐vaccine comparison of T cell epitope coverage for each of the vaccine strains against G4. Vaccine strains are color‐coded and arranged in order following host and respective region, year, and vaccine type. The Y‐axis shows vaccine T cell epitope coverage, shown as a percentage of the maximum score. The horizontal dotted line represents mean of T cell epitope coverage for the strains evaluated in this report. Shaded gray areas the threshold for predicted vaccine efficacy for the US (refer to Table 2 footnote) and EU (Table 4 footnote). Only HL03 is predicted to be protective against G4 in this analysis. Statistically significant p‐values are labeled for each comparison. Figure S2. Whole proteome EpiCC analysis of EU swine influenza vaccines (A) and human influenza vaccines (B) against circulating IAV strains in respective regions and G4 strains. Circulating IAV strains were arranged according to clade and year. Red boxes showed H1N1 G4 strains. [file IRV-17-e13058-s002.docx]

**
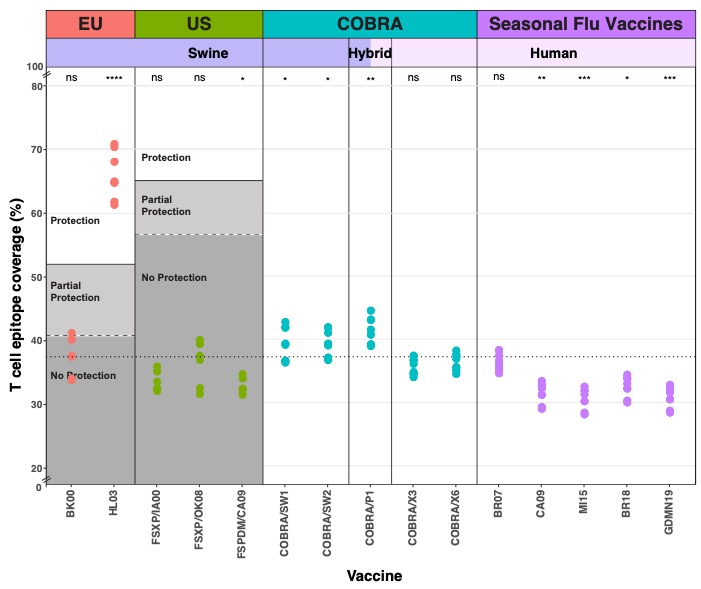
**

**Supplementary Figure 1.** A vaccine-to-vaccine comparison of T cell epitope coverage for each of the vaccine strains against G4. Vaccine strains are color-coded and arranged in order following host and respective region, year, and vaccine type. The Y-axis shows vaccine T cell epitope coverage, shown as a percentage of the maximum score. The horizontal dotted line represents mean of T cell epitope coverage for the strains evaluated in this report. Shaded grey areas the threshold for predicted vaccine efficacy for the US (refer to Table 2 - footnote) and EU (Table 4 - footnote). Only HL03 is predicted to be protective against G4 in this analysis. Statistically significant p-values are labelled for each comparison.

**Supplementary Figure 2.** Whole proteome EpiCC analysis of EU swine influenza vaccines (A) and human influenza vaccines (B) against circulating IAV strains in respective regions and G4 strains. Circulating IAV strains were arranged according to clade and year. Red boxes showed H1N1 G4 strains.
